# Numerical Analysis of Thermophoresis of a Charged Spheroidal Colloid in Aqueous Media

**DOI:** 10.3390/mi12020224

**Published:** 2021-02-23

**Authors:** Yi Zhou, Yang Yang, Changxing Zhu, Mingyuan Yang, Yi Hu

**Affiliations:** Key Laboratory of High Performance Ship Technology, Ministry of Education, School of Energy and Power Engineering, Wuhan University of Technology, Wuhan 430063, China; zhouyi@whut.edu.cn (Y.Z.); yang-yang@whut.edu.cn (Y.Y.); zcxwhut@whut.edu.cn (C.Z.); myyang@whut.edu.cn (M.Y.)

**Keywords:** thermophoresis, thermodiffusion coefficient, spheroids, rods, hydrodynamic effect, particle curvature’s effect

## Abstract

Thermophoresis of charged colloids in aqueous media has wide applications in biology. Most existing studies of thermophoresis focused on spherical particles, but biological compounds are usually non-spherical. The present paper reports a numerical analysis of the thermophoresis of a charged spheroidal colloid in aqueous media. The model accounts for the strongly coupled temperature field, the flow field, the electric potential field, and the ion concentration field. Numerical simulations revealed that prolate spheroids move faster than spherical particles, and oblate spheroids move slower than spherical particles. For the arbitrary electric double layer (EDL) thickness, the thermodiffusion coefficient of prolate (oblate) spheroids increases (decreases) with the increasing particle’s dimension ratio between the major and minor semiaxes. For the extremely thin EDL case, the hydrodynamic effect is significant, and the thermodiffusion coefficient for prolate (oblate) spheroids converges to a fixed value with the increasing particle’s dimension ratio. For the extremely thick EDL case, the particle curvature’s effect also becomes important, and the increasing (decreasing) rate of thermodiffusion coefficient for prolate (oblate) spheroids is reduced slightly.

## 1. Introduction

Thermophoresis describes the motion of a charged colloidal particle with respect to the aqueous media in response to an external applied temperature gradient [1,2,3]. The thermophoresis approach has been used to separate, trap, and concentrate nanoparticles in liquid media [4,5,6,7]. For biological and biocompatible compounds [8] in aqueous solutions, the thermophoretic techniques provide useful tools to quantify the protein−ligand binding constants [9] and the copy numbers of noncoding RNA [10], examine the hydration layer [11], and determine the biomolecular interactions [12].

Extensive theoretical [13,14,15] and experimental studies [16,17,18] on thermophoresis of spherical particles in aqueous solutions have investigated various effects of the particle size [19,20,21], electrolyte concentrations [22,23] and types [24], the bulk temperature [25], and so on. However, as most biological and biocompatible compounds in aqueous solutions are non-spherical [8], the interest in colloidal thermophoresis of non-spherical particles is considerably increasing. Our group [26] carried out thermophoretic experiments of peanut-like particles, and observed both translational and rotational movements with the smaller translational thermophoretic velocities than those of the spherical particles of similar sizes. Several studies [27,28,29,30] have focused on the thermophoresis of rod-like particles. Blanco et al. [27] conducted thermophoretic experiments of the rod-like *fd*-Y21M virus, and they did not find the shape anisotropy of the *fd*-Y21M virus. Tan et al. [28] built a numerical thermophoretic model for charged rods by using the molecular dynamics simulation method, and they constructed the rod with a “shish-kebab” model as connecting beads in a linear disposition. Interestingly, the shape anisotropy of the rod-like particles was found in Tan et al.’s work [28]. Moreover, Wang’s studies [29,30] also investigated the thermophoresis of the rod-like *fd*-virus, and found that the thermodiffusion coefficient $D_{T}$ of the bare-virus increases strongly with the Debye length, whereas the $D_{T}$ of the grafted-virus is almost independent of the Debye length. Based on the Dhont–Briel’s model [31] for spherical colloids, Wang et al. [29,30] proposed a theory that the $D_{T}$ of a rod can be approximated as that of a string of spherical beads, which is proportional to the rod-core dimensions $L/2a_{c}$, with L and $a_{c}$ respectively being the rod contour length and the rod radius. However, in these thermophoretic experiments of rod-like particles [27,29,30], there is one unique particle shape and size, and the Debye length is close to the rod radius.

The aim of this study was to determine the thermodiffusion coefficient of non-spherical particles with an arbitrary electric double layer (EDL) thickness and different particle shapes. We chose spheroidal particles [32] because rod-like colloids such as the *fd*-Y21M virus can be modelled as prolate spheroids, and dish-like colloids such as the red blood cell [33] can be modelled as oblate spheroids. The paper is organized as follows: Section 2 proposes a mathematical model to investigate the particle shape effect on thermophoresis. Section 3 first presents the flow fields around spheroidal particles, and then discusses the dependencies of thermodiffusion coefficient $D_{T}$ (or thermodiffusion coefficient ratio ξ) on the particle’s dimension ratio b/a of the major semiaxis to the minor semiaxis and the ratio κa of the particle’s minor semiaxis to the EDL thickness. Finally, the main conclusions are presented in Section 4.

## 2. Mathematical Model

**Figure 1:**
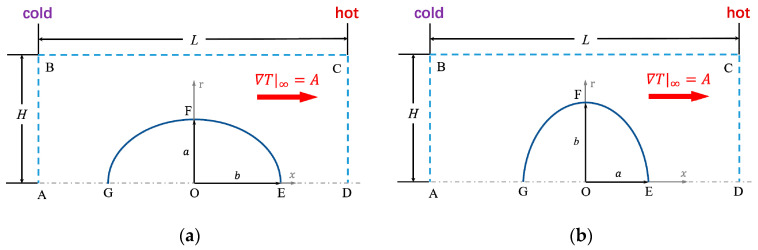
Thermophoresis of a charged spheroidal particle: (**a**) the prolate spheroidal particle with the major semiaxis *b* along the external applied temperature gradient A; (**b**) the oblate spheroidal particle with the minor semiaxis a along the external applied temperature gradient A. With the center of the spheroidal particle located at the origin (O) of a rectangular domain ABCD with dimensions of L×H, the symmetric axis AD is along the external applied temperature gradient A. Boundaries AB and CD are set as the cold and hot sides, respectively, with the lengths of L and H being chosen as more than 200 times the minor semiaxis a, to avoid the boundaries’ effect on the thermophoresis. Boundary BC is the imagined boundary set in the far-field.

We consider the thermophoretic motion of a dilute spheroidal particle suspension. Due to negligible particle–particle interactions, a single charged spheroid motion in the electrolyte solution is studied herein. Driven by the external applied temperature gradient A, the spheroidal particle moves to the cold/hot side [34] with the steady-state thermophoretic velocity $u_{TP}$. With setting the symmetric axis along the external applied temperature gradient A, the three-dimensional problem is simplified to axially symmetric cylindrical coordinates (r,x), as shown in Figure 1. When the major semiaxis b is along the symmetric axis, the spheroidal particle is prolate, as in Figure 1a; when the minor semiaxis a is along the symmetric axis, the spheroidal particle is oblate, as in Figure 1b.

### 2.1. Governing Equations

As has been discussed in the literature [14], the temperature distributions are governed by the energy equation as

(1) $$k_{p}\nabla^{2}T_{p}=0\;\mathrm{for}\;\mathrm{the}\;\mathrm{particle},$$

(2) $$\rho_{f}c_{f}V\cdot \nabla T_{f}=k_{f}\nabla^{2}T_{f}\;\mathrm{for}\;\mathrm{the}\;\mathrm{fluid},$$

where $k_{p}$ and $k_{f}$ are the thermal conductivities of particle and fluid, respectively. $\rho_{f}$ is the fluid density and $c_{f}$ is the fluid heat capacity. The subscripts p and f represent the particle and fluid regions, respectively. The nomenclature is provided in Section S1 of supporting materials.

In the particle-fixed reference frame, the spheroid particle is fixed to be static, and its surrounding electrolyte solution flows in the opposite direction of the thermophoretic motion, with the fluid velocity field being denoted as V. The unknown steady-state thermophoretic velocity is opposite to the far-field fluid velocity as $u_{TP}=-u_{\infty }$. The flow of the electrolyte solution is governed by the Navier–Stokes equations as

(3) ∇⋅V=0,

(4) $$\rho V\cdot \nabla V=-\nabla p+\mu \nabla^{2}V+\rho_{e}E-\frac{1}{2}E^{2}\nabla \varepsilon ,$$

where the fluid velocity $V=\left(V_{r},V_{x}\right)$ with $V_{r}$ and $V_{x}$ as radial and axial velocities, respectively, and p is the fluid pressure. The dynamic viscosity and the fluid permittivity are assumed to be the same as those of water, with the expressions respectively being $\mu =2.761\times 10^{-6}\exp\left(1713/T\right)$ and $\varepsilon =305.7\times 8.85\times 10^{-12}\exp\left(-0.004T\right)$ [14]. In Equation (4), the last two terms on the right-hand side are the electric body force and the dielectrophoretic force acting on the fluid, where $\rho_{e}$ is the free electric charge density, and E is the local electric field. It is known that the free charge density is defined as

(5) $$\rho_{e}=F\sum_{i=1}^{N}z_{i}c_{i},$$

where N is the total number of ion species in the solution; the Faraday constant F=96485.34  C/mol; $z_{i}$ and $c_{i}$ are the *i*^th^ (i=1 for cations and i=2 for anions) ion valence and molar concentration, respectively. The *i*^th^ ion molar concentration is governed by the Nernst–Planck equation as

(6) $$\nabla \cdot \left(Vc_{i}-D_{i}\nabla c_{i}+\frac{z_{i}Fc_{i}D_{i}}{RT}E\right)=0,$$

where the gas constant R=8.31J/mol⋅K. According to Einstein’s law, the *i*^th^ ion mass diffusivity $D_{i}$ is given as $D_{i}=k_{B}T/\left(6\pi \mu a_{i}\right)$, with the Boltzmann constant $k_{B}=1.38\;\times \;10^{-23}J/K$ and $a_{i}$ as the *i*^th^ ion radius. The first term $Vc_{i}$ in the parentheses on the left-hand side of Equation (6) is due to the bulk convection; the second term $-D_{i}\nabla c_{i}$ is the diffusional process; the last term $\frac{z_{i}Fc_{i}D_{i}}{RT}E$ is due to the electrical migration. It is known that E is related to the electric potential ϕ as E=−∇ϕ, and ϕ is related to $\rho_{e}$ through the Poisson equation:

(7) $$-\nabla \cdot \left(\varepsilon \nabla \phi \right)=\rho_{e}.$$

Equations (1)–(7) form a closed set of governing equations for the thermophoresis of spheroids in the aqueous solution. They can be applied to describe the temperature distribution, the solution flow, the ion concentration distribution, and the electric potential distribution in thermophoresis under various conditions. The corresponding dimensionless forms of these equations are written as

(8) $$\nabla^{*}{}^{2}\Theta_{p}=0,$$

(9) $$Pe_{t}V^{*}\cdot \nabla^{*}\Theta_{f}=\nabla^{*}{}^{2}\Theta_{f},$$

(10) $$\nabla^{*}\cdot V^{*}=0,$$

(11) ReV*⋅∇*V*=−∇*p*+μ*∇*2V*+fref1∇*ϕ*∑i=1Nzici*+fref2(∇*ϕ*)2∇*ε*,

(12) $$\nabla^{*}\cdot \left(Pe_{c}V^{*}c_{i}{}^{*}-D_{i}{}^{*}\nabla^{*}c_{i}{}^{*}-\frac{z_{i}c_{i}{}^{*}}{\Theta }D_{i}{}^{*}\nabla^{*}\phi^{*}\right)=0,$$

(13) $$-\nabla^{*}\cdot \left(\varepsilon^{*}\nabla^{*}\phi^{*}\right)=\frac{1}{2}{\left(\kappa_{ref}a\right)}^{2}\sum_{i=1}^{N}z_{i}c_{i}{}^{*},$$

The dimensionless variables for the gradient operator, the temperature, the velocity vector, the pressure, the *i*^th^ ion concentration, and the electric potential are defined respectively as follows:

(14) $$\nabla^{*}=a\nabla ,\;\Theta =\frac{T}{T_{0}},\;V^{*}=\frac{V}{u_{ref}},\;p^{*}=\frac{p}{p_{ref}},\;c_{i}{}^{*}=\frac{c_{i}}{c_{0}},\;\phi^{*}=\frac{\phi }{\phi_{ref}},$$

and the reference parameters for the velocity, the pressure, and the electric potential are defined respectively as

(15) $$u_{ref}=\left(1-\frac{d\ln\varepsilon }{d\ln T}\right)\frac{\varepsilon_{ref}\zeta^{2}}{12\mu_{ref}T_{0}}A,\;p_{ref}=\frac{u_{ref}\mu_{ref}}{a},\;\phi_{ref}=\frac{RT_{0}}{F},$$

where a is the spheroidal minor semiaxis; $T_{0}$ is the average temperature of the particle; $u_{ref}$ is the thermophoretic velocity under the external applied temperature gradient A for the extremely thin EDL case [2]. ζ is the particle zeta potential, and $c_{0}$ is the cation/anion concentration in the bulk region for the symmetric electrolyte. $\kappa_{ref}a$ is the ratio of the particle’s minor semiaxis a to the reference EDL thickness $\kappa_{ref}{}^{-1}$, with $\kappa_{ref}=\sqrt{2c_{0}F^{2}/\varepsilon_{ref}RT_{0}}$. The dimensionless thermophysical parameters for the fluid viscosity, the electrical permittivity, and the mass diffusivity are introduced as

(16) $$\mu^{*}=\frac{\mu }{\mu_{ref}},\;\varepsilon^{*}=\frac{\varepsilon }{\varepsilon_{ref}},\;D_{i}{}^{*}=\frac{D_{i}}{D_{ref}},$$

where the subscript ref represents the reference parameters at the average temperature of particle $T_{0}$, and the reference mass diffusivity $D_{ref}=1\times 10^{-9}m^{2}/s$. The characteristic numbers of the thermal Peclet number, the ion Peclet number, and the Reynolds number are given as

(17) Pet=urefaα, Pec=urefaD0, Re=ρurefaμref,

with α as the fluid thermal diffusivity. Furthermore, the dimensionless body force coefficients for the electric body force and the dielectrophoretic force are respectively expressed as

(18) $$f_{ref1}=-\frac{Fc_{0}\phi_{ref}a}{u_{ref}\mu_{ref}},\;f_{ref2}=-\frac{\varepsilon_{ref}\phi_{ref}{}^{2}}{2u_{ref}\mu_{ref}a}.$$

### 2.2. Boundary Conditions

For the applied temperature field, we impose a constant temperature gradient along the *x* direction through setting the non-dimensional values of $1-AaL^{*}/2T_{0}$ at the cold side and $1+AaL^{*}/2T_{0}$ at the hot side. For the flow field, we employ a non-slip boundary condition at the particle–liquid interface, and far-field boundary conditions of zero shear stress and zero pressure along the imagined boundaries. For the ion concentration field, the zero-ion penetration boundary condition is set at the particle–liquid interface, and a dimensionless value of 1 is set along the imagined boundaries. For the electric potential field, we impose a constant value of $\zeta^{*}=\zeta /\phi_{ref}$ at the particle–liquid interface, and insulation conditions along the imagined boundaries. The detailed boundary conditions are provided in Section S2 of Supplementary Materials.

### 2.3. Numerical Method

Equations (8)–(13), which are strongly coupled, were numerically solved with the commercial software COMSOL Multiphysics 5.6. To verify the numerical method, we simulated a benchmark analytical solution of thermophoresis of a single spherical particle wherein the thermal conductivities of particle and liquid were the same, and found that the numerical results are in excellent agreement with the benchmark solution [35]; the detailed description is in Section S3 of the supporting materials.

## 3. Results and Discussion

In the present work, the thermophoresis of the charged spheroid worked at room temperature (i.e., $T_{0}=298.15K$), and it was driven by the external applied temperature gradient, which was set as the experimental value $A=1.55\times 10^{4}K/m$ [21]. The aqueous media was chosen as a lithium chloride (LiCl) solution with a negligible thermoelectricity effect [36]. The thermal conductivity of particle was assumed to be the same as that of fluid to simplify the analysis.

### 3.1. Flow Field Around Spheroidal Particles

**Figure 2:**
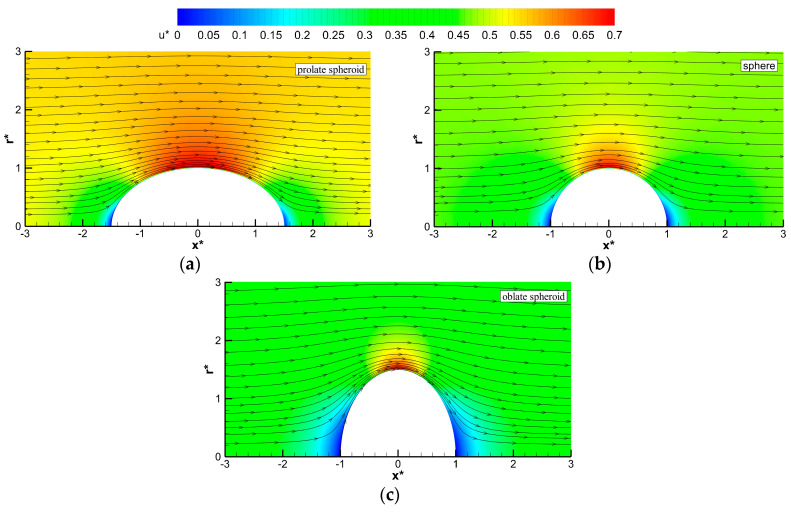
Distributions of the dimensionless axial velocity around the particles when κa=100 for different spheroidal particles: (**a**) prolate spheroids with b/a=1.5, (**b**) sphere with b/a=1, and (**c**) oblate spheroids with b/a=1.5. The solid lines with arrows denote the streamlines.

Within the particle-fixed reference frame, the particle is fixed and the fluid flows in a direction opposite to the particle thermophoretic motion. For the case wherein the EDL thickness is much smaller than the particle’s minor semiaxis (e.g., κa=100), the fluid flow fields around a prolate spheroid, a spherical particle, and an oblate spheroid are presented in Figure 2. It is shown that the fluid around these spheroids flows along the x direction (i.e., the temperature gradient direction). Due to the particle curvature, the streamline curvature around the prolate spheroid is lower than that around the spherical particle, and the streamline curvature around the oblate spheroid becomes higher. Moreover, the velocity magnitudes at the upper pole of the particle surface ($\left(x^{*},r^{*}\right)=\left(0,1\right)$ for prolate spheroids and $\left(x^{*},r^{*}\right)=\left(0,b/a\right)$ for oblate spheroids) increase sharply near the particle surface, and then decrease slowly as one gets further from the particle surface. The magnitudes of the far-field velocity $u_{\infty }$ of the prolate spheroid are much larger than that of the spherical particle, and the magnitude of $u_{\infty }$ of the oblate spheroid is the smallest.

**Figure 3:**
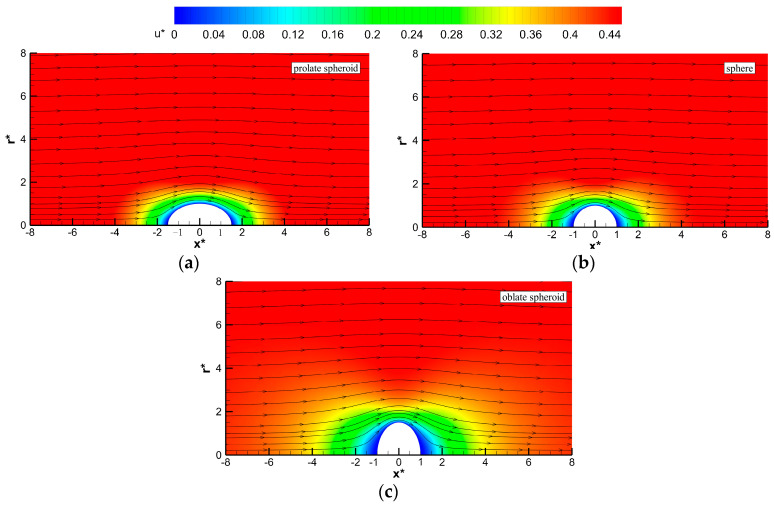
Distributions of the dimensionless axial velocity around the particles when κa=0.01 for different spheroidal particles: (**a**) prolate spheroids with *b*/*a* = 1.5, (**b**) sphere with *b*/*a* = 1, and (**c**) oblate spheroids with *b*/*a* = 1.5. The solid lines with arrows denote the streamlines.

Figure 3 depicts the fluid velocity fields around a spheroid with three different particle shapes, when the EDL is thick (e.g., κa=0.01). Compared with Figure 2, it is clearly shown that the variation regions of flow fields of these spheroids for thick EDL cases are larger than those for thin EDL cases; the velocity magnitudes increase as the distance from the particle surface increases. Moreover, the magnitude of $u_{\infty }$ of the prolate (oblate) spheroid for thick EDL cases is slightly larger (smaller) than that of the spherical particle.

### 3.2. Thermodiffusion Coefficient of Spheroidal Particles

The thermodiffusion coefficient $D_{T}$ is proportional to the far-field fluid velocity $u_{\infty }$ through the following expression.

(19) $$D_{T}=\frac{u_{\infty }}{A}.$$

**Figure 4:**
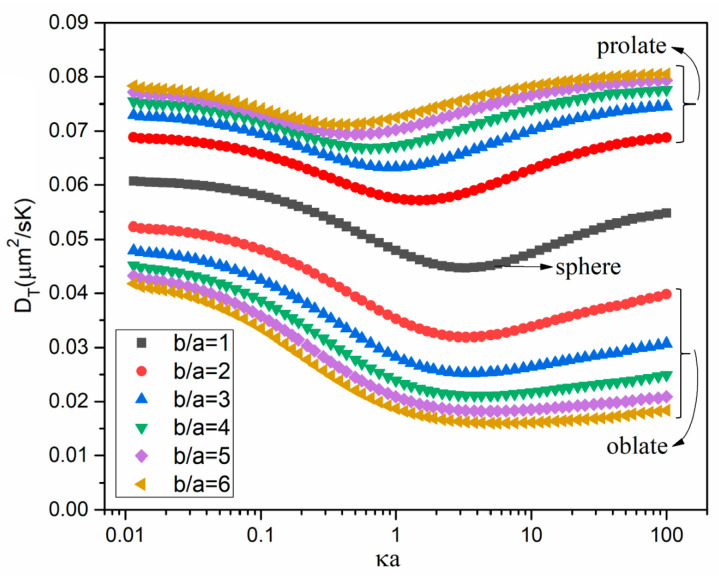
Dependence of the numerically computed thermodiffusion coefficient $D_{T}$ on the ratio κa of the particle’s minor semiaxis to the EDL thickness at the average temperature $T_{0}$ for six different values of the particle’s dimension ratio b/a for both prolate and oblate spheroids. b/a=1 represents the sphere.

Figure 4 displays how the thermodiffusion coefficients $D_{T}$ of prolate spheroids vary with κa for b/a ranging from 1 to 6. It is shown that the thermodiffusion coefficient of prolate spheroids is larger than the spherical particle, which is similar to the conclusion of [29]. The thermodiffusion coefficient variation of prolate spheroids with κa is similar to that of the spherical particle, showing a decreasing and then increasing trend. With the increase of b/a, the prolate particle approaches a rod-like particle, and the thermodiffusion coefficient increases. Due to the faster increasing rate, the $D_{T}$ of prolate spheroids for thin EDL cases becomes larger than those for thick EDL cases, and κa corresponding to the turning point of $D_{T}$ reduces for a larger b/a.

The thermodiffusion coefficient variation of oblate spheroids with κa is also shown in Figure 4. We can see that the thermodiffusion coefficient of oblate spheroids is smaller than the spherical particle, with a similar dependence of $D_{T}$ on κa. With the increase of b/a, the oblate particle approaches a disk-like particle in shape, and the thermodiffusion coefficient decreases. Due to the faster decreasing rate, the $D_{T}$ of oblate spheroids for thin EDL cases become much smaller than those for thick EDL cases, and the $D_{T}$ of b/a=6 almost keeps a fixed value when κa>3.

### 3.3. Thermodiffusion Coefficient Ratio of Spheroids to Spheres

**Figure 5:**
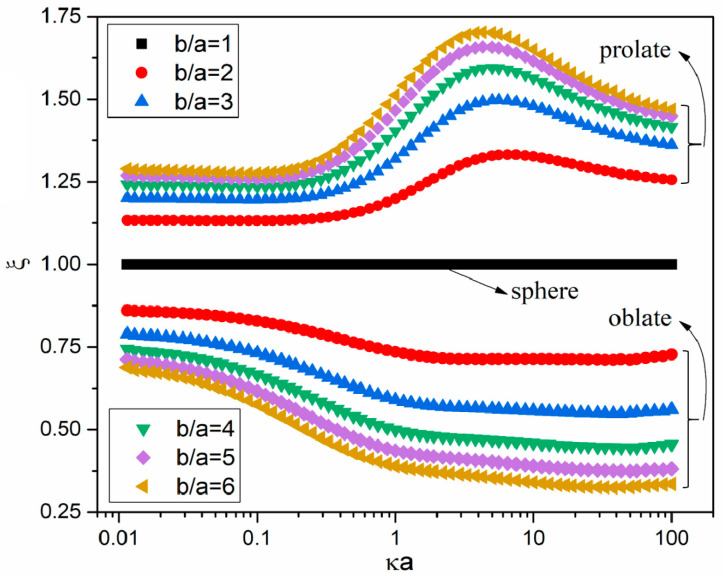
Dependence of the thermodiffusion coefficient ratio $\xi =D_{T}/{D_{T}|}_{S}$ on κa for six different values of the particle’s dimension ratios b/a.

To more clearly show the particle shape effect on thermodiffusion coefficients $D_{T}$, the thermodiffusion coefficient ratio $\xi =D_{T}/{D_{T}|}_{S}$ is discussed (here, ${D_{T}|}_{S}$ is the thermodiffusion coefficient of a spherical particle). Figure 5 shows the variations of the numerical ξ with κa for b/a ranging from 1 to 6. For thick EDL cases (κa<0.1), ξ of prolate particles is independent of κa. With increasing κa, ξ of prolate spheroids increases to a maximum value and then decreases. However, ξ of oblate spheroids for thick EDL cases decreases with κa, and this decreasing tendency becomes stronger for a larger b/a. With an increasing κa, the ξ of oblate spheroids is almost independent of κa for thin EDL cases.

#### 3.3.1. Thermodiffusion Coefficient Ratio for the Extremely Thin EDL Case

**Figure 6:**
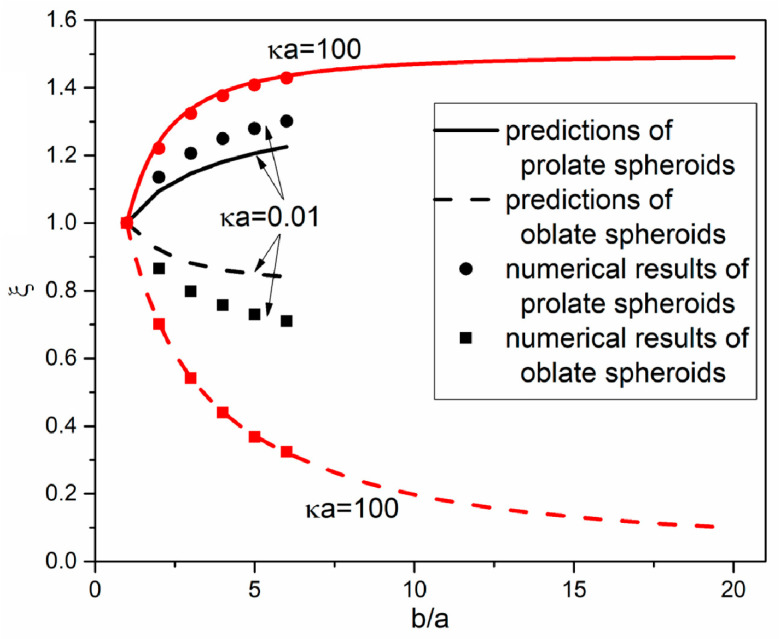
Comparison of thermodiffusion coefficient ratios ξ among the numerically computed results and the results calculated from Equations (20) to (23) for prolate and oblate particles, when *b*/*a* is in the range of 1 to 20 under two values of κa (κa=0.01 and 100).

For the extremely thin EDL case (i.e., κa=100), the particle curvature’s effect on the ion distribution is negligible, and the ions move relatively with respect to a planar surface [37]. Moreover, according to our previous work [14], the thermophoretic velocity is proportional to the local temperature gradient at the upper pole of the particle surface ($\left(x^{*},r^{*}\right)=\left(0,1\right)$ for prolate spheroids and $\left(x^{*},r^{*}\right)=\left(0,b/a\right)$ for oblate spheroids). Therefore, the thermophoretic problem for the extremely thin EDL case can be treated as a thermal creep flow around the particle surface, and the thermophoretic force $F_{TP}$ is balanced by the viscous drag [38]. Hence, the thermodiffusion coefficient ratio for a prolate spheroid for the extremely thin EDL case is given as

(20) $$\xi =1.5\frac{b}{a}\frac{1+\left(\frac{1}{\lambda }-\lambda \right)\mathrm{arccoth}\lambda }{\sqrt{\lambda^{2}-1}\left[\frac{\lambda }{\lambda^{2}-1}-\frac{1}{\lambda }\right]},$$

and the thermodiffusion coefficient ratio for an oblate spheroid under the extremely thin EDL case is given as

(21) $$\xi =1.5\frac{a}{b}\frac{1-\left(\bar{\lambda }+\frac{1}{\bar{\lambda }}\right)\mathrm{arccot}\bar{\lambda }}{\sqrt{1+{\bar{\lambda }}^{2}}\left(\frac{\bar{\lambda }}{1+{\bar{\lambda }}^{2}}-\frac{1}{\bar{\lambda }}\right)},$$

where $\lambda =b/\sqrt{\left(b^{2}-a^{2}\right)}$ and $\bar{\lambda }=a/\sqrt{\left(b^{2}-a^{2}\right)}$. The estimations of ξ for prolate and oblate spheroids expressed by Equations (20) and (21) with the particle’s dimension ratio b/a of the major semiaxis to the minor semiaxis for the extremely thin EDL case are shown in Figure 6. Clearly, our analytical results are in the remarkably good agreement with our numerical results for both prolate and oblate spheroids. Therefore, one can conclude that for the extremely thin EDL case, the thermophoresis of spheroids is mainly under the hydrodynamic effect, and the particle curvature’s effect on the ion distribution is negligible. It is shown in Figure 6 that the thermodiffusion coefficient ratio ξ for a prolate (an oblate) spheroid is larger (smaller) than one, and it increases (decreases) with b/a. With a further increasing b/a, the ξ of prolate spheroids increases and converges to a fixed value of 1.5, which means that the thermodiffusion coefficient of a rod-like particle for the extremely thin EDL case is 1.5 times that of a spherical particle. ξ for oblate spheroids becomes close to zero with a further increasing b/a, as the flow is blocked by the dish-like particle. For human red blood cells with a b/a of 4, the thermodiffusion coefficient for the extremely thin EDL cases is about 0.4 times of that of spherical particles.

#### 3.3.2. Thermodiffusion Coefficient Ratio for the Extremely Thick EDL Case

For the extremely thick EDL case (i.e., κa=0.01), the EDL region is much larger than the flow region as shown in Figure 3, which means that the particle curvature’s effect on the ion distribution is noticeable. According to [39], the Stokes drag on prolate and oblate spheroids is proportional to the thermophoretic velocity, and it is balanced by the thermophoretic force $F_{TP}$. The thermodiffusion coefficient ratio of prolate spheroids is given as

(22) $$\xi =\frac{3}{8}\frac{F_{TP}}{{F_{TP}|}_{s}}\frac{\left(\left({1+e}_{p}{}^{2}\right)L-2e_{p}\right)}{\frac{b}{a}e_{p}{}^{3}},$$

and the thermodiffusion coefficient ratio of oblate spheroids is given as

(23) $$\xi =\frac{3}{8}\frac{F_{TP}}{{F_{TP}|}_{s}}\frac{e_{p}{\left(1-e_{p}{}^{2}\right)}^{\frac{1}{2}}-\left(1-2e_{p}{}^{2}\right)\arcsin e_{p}}{\frac{b}{a}e_{p}{}^{3}},$$

where the eccentricity $e_{p}=\sqrt{b^{2}-a^{2}}/b$, and the thermophoretic force $F_{TP}$ is obtained through integrating the numerical thermophoretic force density (the electrical force density and the dielectrophoretic force density of Equation (11)) in the EDL region.

The variations of thermodiffusion coefficient ratios of prolate and oblate spheroids with b/a for the extremely thick EDL case (i.e., κa=0.01) are also shown in Figure 6. Clearly ξ of prolate (oblate) spheroids for κa=0.01 have a similar increasing (decreasing) trend with b/a as that for κa=100, meaning that the hydrodynamic effect is also significant for the thick EDL cases. However, the value of ξ of prolate (oblate) spheroids for κa=0.01 is smaller (larger) than that for κa=100, which indicates that the particle curvature’s effect on the ion distribution retards the increasing (decreasing) trend. The semi-analytical results are also shown in Figure 6, which reasonably agree with the numerical results. The deviation of our predictions from the numerical results is probably due to the ignored temperature gradient when calculating the Stokes drag. Therefore, for the thermophoresis of spheroids under thick EDL cases, both the hydrodynamic effect and the particle curvature’s effect on the ion distribution are significant.

The present work describes the thermophoresis of prolate and oblate spheroids in aqueous media, showing the anisotropic thermophoretic motion. The major semiaxis of the prolate spheroid is parallel to the external temperature gradient A, while the major semiaxis of the oblate spheroid is perpendicular to A. According to the [28], a freely moving spheroid is randomly aligned in the liquid, with the unique thermodiffusion factor $D_{T,iso}=\left(2D_{T,⊥}+D_{T,∥}\right)/3$, where $D_{T,⊥}$ and $D_{T,∥}$ are, respectively, the thermodiffusion coefficients of spheroids with the major semiaxis being perpendicular and parallel to A. In the present work, thermodiffusion coefficient variation studies of prolate and oblate spheroids with κa and b/a can shed light on the variations of $D_{T,∥}$ and $D_{T,⊥}$. Due to the hydrodynamic effect, when the major semiaxis is parallel (perpendicular) to A, the thermodiffusion coefficient of the spheroid is larger (smaller) than that of the sphere (i.e., $D_{T,∥}>{D_{T}|}_{S},\;D_{T,⊥}<{D_{T}|}_{S}$). Especially for thin EDL cases, when the particle’s dimension ratio b/a becomes large, $D_{T,∥}>>D_{T,⊥}$. Therefore, the unique thermodiffusion factor of a freely moving spheroid can be simplified as $D_{T,iso}=D_{T,∥}/3$ for thin EDL cases, when b/a>>1.

The present work shows that the thermophoresis of symmetric spheroids in aqueous media has a translational motion. If the particle becomes asymmetric, e.g., the peanut-like particle [26] and the Janus particle [40], a rotational thermophoresis can be observed. Such anisotropic thermophoresis of an asymmetric particle could have applications in micro- and nano-swimmers [41].

## 4. Conclusions

We have developed a numerical model for describing the thermophoresis of a single charged spheroid in aqueous media. The numerical results show that the thermophoretic coefficient $D_{T}$ of prolate (oblate) spheroids is larger (smaller) than that of spherical particles, and the increasing (decreasing) tendency is dependent on κa and b/a. For the extremely thick EDL case (i.e., κa=0.01), under both the hydrodynamic effect and the particle curvature’s effect, the increasing (decreasing) rate of $D_{T}$ for prolate (oblate) spheroids is smaller than that for the extremely thin EDL case. For the extremely thin EDL case (i.e., κa=100), due to the dominant hydrodynamic effect, the thermophoretic coefficient $D_{T}$ of prolate (oblate) spheroids increases (decreases) with increasing b/a and converges to a fixed value. When the EDL thickness is close to the particle’s minor semiaxis, the increasing rate of prolate spheroids becomes larger than that for κa=100, but the decreasing rate of oblate spheroids is close to that for κa=100. For a freely moving spheroid, the unique thermodiffusion factor $D_{T,iso}$ is one third of the thermodiffusion coefficient $D_{T,∥}$ of spheroids, with the major semiaxis being parallel to the external temperature gradient A, for thin EDL cases when b/a>>1.

## Supplementary Materials

The following are available online at https://www.mdpi.com/2072-666X/12/2/224/s1. S1: Nomenclature. S2: Boundary conditions of the numerical model. S3: Validation of the numerical model.
