# Supplementary material for: Numerical Analysis of Thermophoresis of a Charged Spheroidal Colloid in Aqueous Media
Source: Micromachines (Basel). 2021 Feb 23;12(2):224. doi: 10.3390/mi12020224 (PMC7926884; doi:10.3390/mi12020224)
Supplement: Supplementary file 1 [file micromachines-12-00224-s001.pdf]

Supporting materials for "Numerical analysis of thermophoresis of a charged spheroidal colloid in aqueous media"

Yi Zhou[1], Yang Yang[1], Changxing Zhu[1], Mingyuan Yang[1] and Yi Hu[1,*]

[1] Key Laboratory of High Performance Ship Technology, Ministry of Education, School of Energy and Power Engineering, Wuhan University of Technology, Wuhan 430063, PR China

* Correspondence: huyi75@whut.edu.cn; Tel.: +86-27-8654-0330

S1 Nomenclature

$\boldsymbol{A}$	Externally applied temperature gradient [K/m]
a	Spheroidal minor semiaxis [m]
a_c	Rod radius [m]
b	Spheroidal major semiaxis [m]
c	Particle molar concentration [mol/m^3]
	Heat capacity [$\mathrm{J/(kg \cdot K)}$]
D	Particle mass diffusivity [m^2/s]
D_T	Thermodiffusion coefficient [$\mathrm{m^2/s \cdot K}$]
$\boldsymbol{E}$	Local electric field [V/m]
e_p	Eccentricity [1]
$\boldsymbol{f}$	Force density [N/m^3]
F	Faraday constant, 96,485.34 C/mol
F_{TP}	Thermophoretic force [N]

H	Width of temperature gradient zone [m]
k	Thermal conductivity [$\mathrm{W/m \cdot K}$]
k_B	Boltzmann constant, $1.38\times10^{-23}\ \mathrm{J/K}$
L	Rod contour length [m]
	Length of temperature gradient zone [m]
N	Total number of ion species [1]
$\boldsymbol{n}$	Unit vector outward normal to the corresponding surfaces
r, x	Cylindrical coordinates
p	Fluid pressure [Pa]
Pe_t	Thermal Peclet number [1]
Pe_c	Ionic Peclet number [1]
R	Universal gas constant, 8.314462 $\mathrm{J/(mol \cdot K)}$
Re	Reynolds number [1]
T	Temperature [K]
u, v	Velocities in x, r directions [m/s]
$\mathbf{u}_\infty$	Far-field fluid velocity [m/s]
$\boldsymbol{u}_{TP}$	Thermophoretic velocity [m/s]
$\boldsymbol{V}$	Fluid velocity field [m/s]
z	Valence of ions

Greek symbols

α	Fluid thermal diffusivity [m^2/s]
ε	Fluid permittivity [F/m]
ϕ	Electric potential [V]
Φ	Dimensionless electric potential [1]
∇	Gradient operator [1/m]
∇T	Externally applied temperature gradient [K/m]
κ	Reciprocal of electrical double layer thickness [1/m]
μ	Viscosity [$\mathrm{kg}/(\mathrm{m}\cdot\mathrm{s})$]
ρ	Fluid density [kg/m^3]
ρ_e	Free electric charge density [C/m^3]
Θ	Dimensionless temperature [1]
ξ	Thermodiffusion coefficient ratio [1]
ζ	Particle zeta potential [V]

Superscripts

*	Non-dimensionalized parameter

Subscripts

0	Parameters related to the average value
i	Parameters related to i^{th} ions
f	Parameters related to the fluid
p	Parameters related to the particle

ref	Reference parameters

Abbreviations

EDL	Electrical double layer

S2 Boundary conditions of the numerical model

A prolate spheroidal and an oblate spheroidal particle are illustrated schematically in Figure 1, which are under the external applied temperature gradient $\boldsymbol{A}$ at the average temperature T_0. Here, non-dimensional boundary conditions of the model are listed for the temperature field, the ion concentration field, the electric potential field, and the velocity field, respectively. The boundary conditions along two planes AB and CD are respectively expressed as

$$\Theta_{x^*=-L^*/2} = 1 - AaL^*/2T_0 \text{ on the plane AB, and } \Theta_{x^*=L^*/2} = 1 + AaL^*/2T_0 \text{ on the plane CD.} \quad (1)$$

The symmetry is set along the symmetric axis AD, and the isothermal conditions is set for the imagination boundary BC in the far-field. Both of them are expressed as

$$\boldsymbol{n} \cdot \nabla^* \Theta = 0 \text{ on the planes AD and BC.} \quad (2)$$

where $\boldsymbol{n}$ represents the unit normal vector outward to the corresponding plane, and $\nabla^* = \left(\partial/\partial r^*, \partial/\partial x^*\right)$ is the dimensionless gradient operator.

The segments EF and FG are defined as the particle-fluid interface, and the heat transfer across such particle-fluid interface is expressed as

$$-k_p \boldsymbol{n} \cdot \nabla^* \Theta_p = -k_f \boldsymbol{n} \cdot \nabla^* \Theta_f \text{ and } \Theta_p = \Theta_f \text{ on the segments EF and FG.} \quad (3)$$

With the assumption that planes AB, BC and CD are in the bulk electrolyte solution, the ith ionic concentration along these planes is equal to the bulk electrolyte concentration. The dimensionless i^{th} ionic concentration is expressed as:

$$c_i^* = 1 \text{ on the planes AB, BC and CD.} \quad (4)$$

The boundary conditions for both the particle-liquid interface EFG and the symmetric axis AD are zero ion flux, with the expression given by

$$\boldsymbol{n} \cdot \left(-D_i^* \nabla^* c_i^* - \frac{z_i c_i^* D_i^*}{\Theta} \nabla^* \Phi \right) = 0 \text{ on the segments EF, FG and AD.} \quad (5)$$

Along the particle-fluid interface EFG, the electric potential boundary condition is the constant zeta potential as

$$\Phi = \zeta / \phi_{ref} \text{ on the segments EF and FG.} \quad (6)$$

The boundary conditions for the symmetric axis AD and segments AB, BC, and CD are set as zero charge as

$$\boldsymbol{n} \cdot \left(-\varepsilon^* \nabla^* \Phi \right) = 0 \text{ on the planes AB, BC, AD and CD.} \quad (7)$$

Along the particle-fluid interface EFG, the non-slip boundary condition is set as

$$\boldsymbol{n} \cdot \boldsymbol{V}^* = 0 \text{ on the segments EF and FG.} \quad (8)$$

Along the segments AB, BC and CD, zero shear stress as well as zero pressure are set as

$$\boldsymbol{n}\cdot\nabla^*\boldsymbol{u}^* = 0\,,\ p^* = 0 \text{ on the planes AB, BC and CD} \tag{9}$$

Along the symmetric axis AD, the symmetry is set as

$$\boldsymbol{n}\cdot\nabla^*\boldsymbol{u}^* = 0 \text{ on the plane AD} \tag{10}$$

S3 Validation of the numerical model

To validate our numerical model, the thermophoresis of a spherical particle driven by the externally applied temperature gradient $\boldsymbol{A}$ was simulated, when the thermal conductivity of particle is assumed to be the same as that of liquid. Both the numerical results and the prediction of the literature study are plotted in Figure S1. It was observed that the numerical results for the arbitrary EDL thickness are in excellent agreement with the prediction of Rasuli and Golestanian [35]. Therefore, the numerical model was validated, which was employed to describe the thermophoresis of a single charged spheroid in the aqueous media.

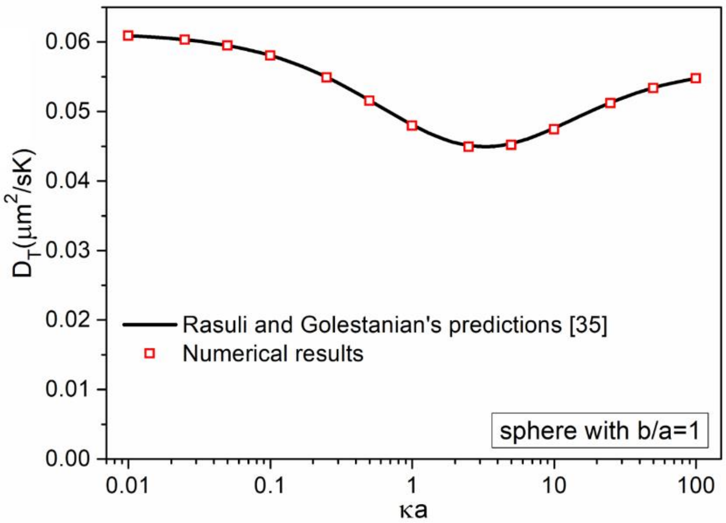

Figure S1. Comparison between the numerical thermodiffusion coefficients D_T and the predictions of literature model [35] for a charged sphere. κa represents the ratio of the particle minor semiaxis to the EDL thickness.
